# The Role of Community Health Professionals in Addressing Intimate Partner Violence: A Scoping Review

**DOI:** 10.1111/phn.70166

**Published:** 2026-08-04

**Authors:** Avanti Adhia, Mary Wingert, Kelly Chadwick, Todd I. Herrenkohl, Paula Kett, Betty Bekemeier

**Affiliations:** 1 Department of Child, Family, and Population Health Nursing, University of Washington, Seattle, Washington, USA; 2 Department of Epidemiology, University of Washington, Seattle, Washington, USA; 3 School of Nursing, University of Washington, Seattle, Washington, USA; 4 Northwest Center for Public Health Practice, University of Washington, Seattle, Washington, USA; 5 School of Social Work, University of Michigan, Ann Arbor, Michigan, USA; 6 Center for Health Workforce Studies, Department of Family Medicine, University of Washington, Seattle, Washington, USA

**Keywords:** capacity building, community health services, intimate partner violence, primary prevention, public health

## Abstract

**Objective:**

Community settings, such as homes, community-based organizations, and schools, are important loci for addressing intimate partner violence (IPV). We identified and synthesized studies on the role of community health professionals in preventing IPV. More specifically, we considered the range and type of interventions used by community health professionals in community settings and whether these interventions appear to be effective in reducing IPV.

**Design:**

We systematically searched four databases for empirical peer-reviewed articles based in the U.S. through August 2024, following the Preferred Reporting Items for Systematic Reviews and Meta-analyses Extension for Scoping Reviews (PRISMA-ScR) guidelines.

**Results:**

We included 45 articles published between 1999 and 2024, spanning quantitative and qualitative study designs. The community health professionals described were most commonly nurses, often delivering home-based interventions focused on perinatal women. Activities related to IPV included providing health education in schools, building community or organizational capacity, providing direct service and case management, and providing coaching and social support to empower individuals.

**Conclusions:**

Community health professionals have an important role in effectively preventing IPV. Additional research is needed to guide these professionals in promoting and implementing a range of IPV interventions in communities, particularly those focused on primary prevention of IPV.

## 1 | Introduction

In the United States (U.S.), over 47% of women and 44% of men report having ever experienced physical violence, sexual violence, or stalking by an intimate partner (Leemis et al. 2022). These experiences of intimate partner violence (IPV), which can also include emotional and psychological abuse, can have severe and long-lasting negative physical health, mental health, and social consequences (Leemis et al. 2022; Stubbs and Szoeke 2022; White et al. 2024). IPV can occur across the lifespan, often starting early in adolescence (Herrenkohl et al. 2022). Among women who experience IPV in their lifetime, more than 70% report their first victimization before age 25 (Leemis et al. 2022). IPV also has significant societal costs, with a population-level economic burden of nearly $3.6 trillion over survivors’ lifetimes in the U.S., including medical costs, lost productivity, and criminal legal system costs (Peterson et al. 2018). Importantly, IPV and its consequences are patterned by structural inequities related to gender, race, socioeconomic status, and geographic location, which shape exposure to IPV and access to safety resources, producing disproportionate harms for marginalized populations (Armstead et al. 2021; Leemis et al. 2022). Given its widespread prevalence and substantial individual and societal consequences, prevention of IPV is needed.

Existing IPV interventions highlight the importance of addressing factors at multiple, reinforcing levels of the social ecology, including the individual, interpersonal/relationships, community, and societal levels (Bacchus et al. 2024; Oram et al. 2022; Trabold et al. 2020). While intervening to support people who have experienced IPV and prevent the recurrence and adverse consequences of violence is critically important, primary prevention (i.e., preventing IPV before it occurs) is vital for assuring measurable reductions in the burden of IPV on communities and society (Graham et al. 2021; Krug et al. 2002; Trabold et al. 2020).

Community settings, such as homes, community-based organizations, and schools, are important loci for IPV prevention and response. Particularly in rural settings, where access to clinical services and healthcare are limited, prevention efforts that engage community members where they live and spend their time is important for reaching beyond the individual to community groups with on-the-ground expertise (Berini et al. 2022; Nam et al. 2025). In these settings, community health professionals (e.g., public health nurses, community health nurses, social workers, health educators, community health workers) are viewed as trusted frontline professionals with a deep understanding of the communities they serve (Jayapaul-Philip et al. 2022). In this community-based context, professionals can build individual- and community-level capacity for proactive prevention efforts through a variety of activities, including psychoeducation, social support, advocacy, coalition-building, and coordinating across sectors (Jayapaul-Philip et al. 2022; Kett et al. 2025). Since the breadth of activities these professionals engage in is wide, systematically identifying specific activities and programs use can inform effective practice. A recent review specific to the role of community health workers in violence prevention identified 20 programs where they engaged in preventing multiple forms of violence (Barbero et al. 2022). This review by Barbero and colleagues highlighted 10 core roles reflected in the activities of community health workers delivering these programs; these roles include offering cultural mediation, social support, capacity building, health education, and direct service provision (Barbero et al. 2022). These workers were also involved in conducting research and evaluation.

In the current scoping review, we build on Barbero’s review by broadening the perspective to a wider range of community health professionals (e.g., including community health workers but also nurses, health educators, social workers, advocates) and focus specifically on IPV prevention and/or response as the focus of the intervention. To inform the development of promising community-based interventions to address IPV, we considered the range and type of interventions used by a variety of community health professionals in community settings and whether these interventions appear to be effective in reducing IPV. We used a scoping review methodology to gain an overview of a broad field, detailing gaps and limitations related to intervention development, testing, and uptake of interventions (Tricco et al. 2018).

## 2 | Methods

### 2.1 | Search Strategy and Eligibility Criteria

For this scoping review, we systematically searched the following databases for empirical peer-reviewed articles based in the U.S. at any time through August 2024: PubMed, Cumulative Index to Nursing and Allied Health Literature (CINAHL), Embase, and Web of Science. We consulted with a research librarian to define a range of search terms for each database, related to concepts of IPV and community health professionals (Table S1). We included terms or geographic filters to filter searches for U.S. location. We also searched the bibliographies of the final included articles for additional references, to screen for inclusion. We followed the Preferred Reporting Items for Systematic Reviews and Meta-analyses Extension for Scoping Reviews (PRISMA-ScR) guidelines (Tricco et al. 2018).

### 2.2 | Article Screening and Selection

We imported all articles from the database searches into Covidence, a web-based collaboration platform for managing reviews (Veritas Health Innovation 2024). Covidence identified duplicate citations for removal. Two independent reviewers screened each title and abstract to assess if it met the following criteria: peer-reviewed journal article (e.g., no conference abstracts, books, grey literature), U.S.-based, published in English, primary research study (e.g., no commentaries), and focused on an IPV prevention and/or response intervention in community settings conducted by community health professionals. After screening titles and abstracts, two independent reviewers reviewed the full text of each screened-in citation to determine final inclusion. Disagreements in article inclusion were discussed among the author reviewers (AA, MW, KC, BB) and resolved with the broader authorship team (TH, PK) when needed.

For our focus on prevention in community settings, we excluded hospitals and law enforcement settings (e.g., police, jails) and focused on settings such as homes, community clinics, health departments, shelters, community-based organizations, and schools. We considered community health professionals to include individuals serving in any of the following positions or roles: public health or community health nurses, social workers, community psychologists, health educators, community health workers, health visitors, doulas, midwives, peer-based support people, community advocates, outreach workers, promotoras, community aides, community health aides, or health aides. If an article reported research on sexual violence or other forms of violence, it was included only if the study related to violence occurring between intimate or dating partners.

### 2.3 | Data Extraction and Synthesis

After articles were screened for inclusion, two independent reviewers extracted the following information for analyses, as available: first author; year of publication; IPV definition; research question; setting; study design; data source; study population; sample size; type/role of community health professional; type of intervention; intervention activities; and major findings. Disagreements in data extraction were resolved through consensus discussion by the study team.

For our presentation of results, we summarized studies using descriptive statistics. Percentages are reported based on article count as the denominator. We also classified each study by type of prevention intervention using the Institute of Medicine’s Classifications for Prevention (Institute of Medicine (US) Committee on Prevention of Mental Disorders 1994). This model classifies interventions into three types: (1) universal, focused on individuals broadly, regardless of IPV risk; (2) selective, focused on individuals at elevated risk of IPV; and (3) indicated, focused on individuals who have experienced IPV.

## 3 | Results

### 3.1 | Search Results

**FIGURE 1 |:**
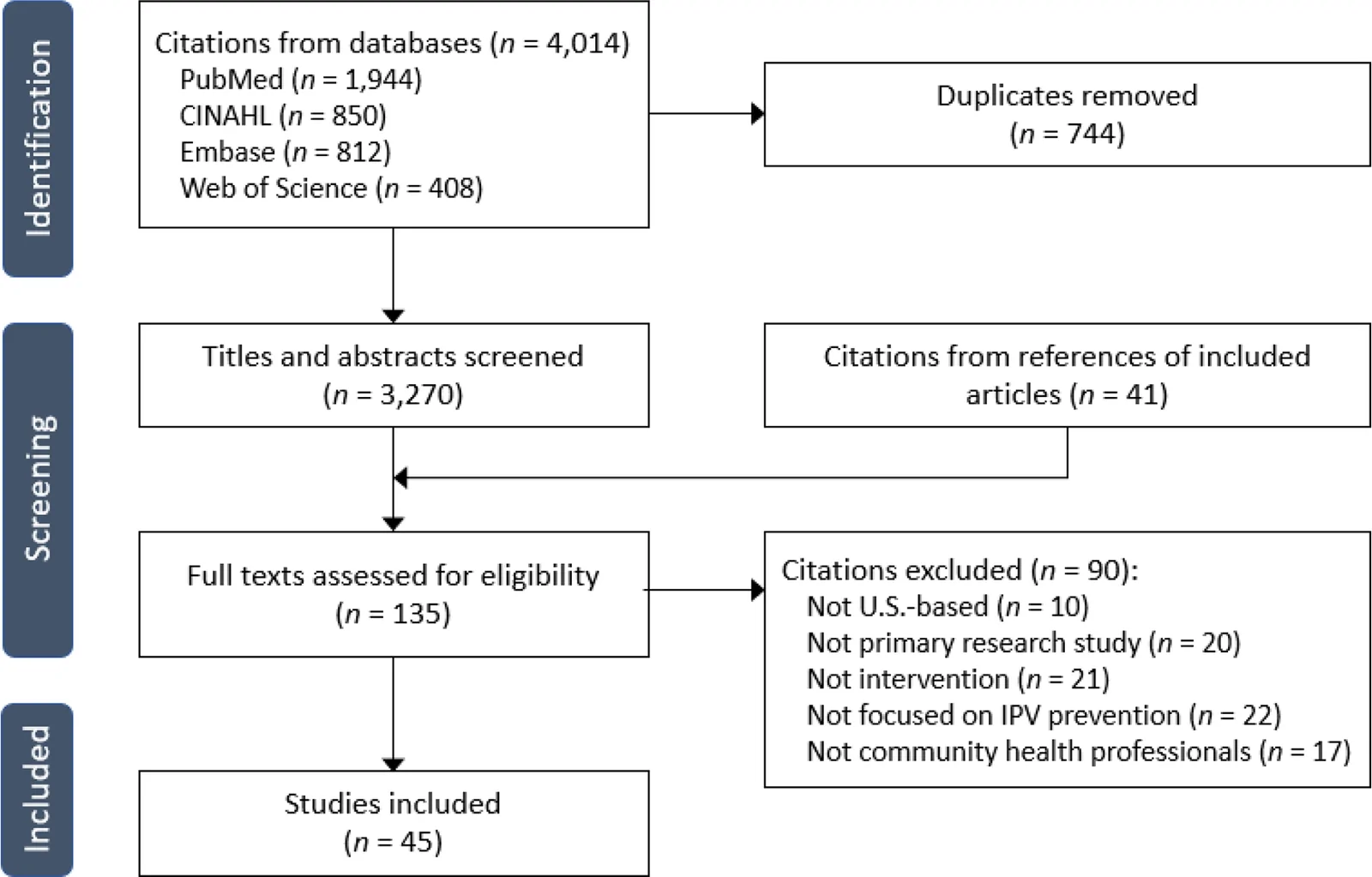
Preferred Reporting Items for Systematic Reviews and Meta-analyses Extension for Scoping Reviews (PRISMA-ScR) flow diagram of search strategy

We identified 4014 articles through searches from the four databases. After importing into Covidence, 744 articles were excluded as duplicates with 3270 articles left to screen. Of these, 3176 were excluded in the title and abstract screening process. We added 41 additional articles to full text screening that were found from the bibliographies of included and fully reviewed articles. In total, we reviewed 135 full texts and excluded 90 of these full text articles, often due to a lack of focus on IPV or lack of an intervention. We included a total of 45 articles for final data extraction and analysis (Figure 1).

Table 1 summarizes study characteristics. The publication span of the 45 included articles was 1999 to 2024, with more than half of the articles (*n* = 24, 53.3%) published between years 2010 and 2019. Approximately two-thirds of studies (*n* = 29, 64.4%) included only quantitative findings (e.g., from randomized controlled trials, pre-post designs, cross-sectional studies), 10 studies (22.2%) included only qualitative findings (e.g., from interviews and focus groups), and 6 studies (13.%) included both data types. Further details of each included study are summarized in Table S2.

### 3.2 | Setting and Community Health Professionals

Over half of studies (*n* = 24, 53.3%) were about preventive interventions that took place in homes, 12 (26.7%) in community clinics, 7 (15.6%) in social service settings (e.g., community-based organizations, shelters), 5 (11.1%) in educational settings, 4 (8.9%) in justice/legal settings, and 4 (8.9%) in other settings (e.g., online, other community venues). Some interventions spanned multiple settings. For example, the IMPACT online training tries to increase the capacity of IPV prevention professionals across settings, including counseling, legal, medical, shelter, and educational settings (Drabkin et al. 2021).

Community health professionals were categorized by type and could be included in multiple categories (e.g., a home visitor who was a nurse was counted in both the nurse and home visitor categories). Nurses were the most commonly specified community health professionals delivering the interventions (*n* = 22 studies, 48.9%). Home visitors were the professionals described as delivering the intervention in 21 studies (46.7%). The home visitors in these 21 studies were comprised of a variety of professional roles, including nurses, paraprofessionals, community health workers, and social workers. Other studies included interventions delivered by staff or advocates from IPV organizations (e.g., rape crisis center educators) (*n* = 12, 26.7%), community health workers (e.g., paraprofessionals, health educators; *n* = 11, 24.4%), and social workers (*n* = 7, 15.6%).

Community health professionals described in the studies engaged in a wide range of activities, including providing health education in schools, building community or organizational capacity, providing direct service and case management, and providing coaching and social support to empower IPV survivors and others at risk of experiencing IPV. Given the multi-faceted nature of the interventions, most studies involved community health professionals serving multiple roles. For example, the Domestic Violence Enhanced Home Visitation Intervention (DOVE) involves perinatal home visitors screening for IPV and leading discussions using a brochure about IPV health consequences, risk factors for homicide, safety planning, and community resources (Bacchus et al. 2016).

### 3.3 | Interventions, Populations, and Classifications for Prevention

The IPV prevention and/or response interventions were designed for multiple populations. Over half of studies (*n* = 24, 53.3%) were interventions for perinatal women, 8 (17.8%) were for IPV survivors, 5 (11.1%) were for at-risk individuals (e.g., low-income and/or immigrant women, Native American communities), 4 (8.9%) were for students, 3 (6.7%) were for community health professionals, and one (2.2%) was for individuals who had perpetrated IPV.

Seven studies (15.6%) included a universal intervention intended for an entire population group regardless of IPV risk or experience (Table 2). Four of these seven studies (57.1%) focused on building organizational capacity to address IPV. The Domestic Violence Prevention Enhancements and Leadership Through Alliance (DELTA) program is a CDC program focused on primary prevention of IPV through funding state coalitions to engage in statewide primary prevention efforts and to provide training, technical assistance, and financial support to local communities (Cox et al. 2010). The IMPACT intervention is an online training to increase capacity of IPV prevention professionals to effectively implement evidence-based IPV prevention programs by increasing knowledge and skills (e.g., emotion regulation, conflict resolution) (Drabkin et al. 2021). The Domestic Violence and Health Care Partnerships (DVHCP) program promotes collaboration between IPV advocates and health care sites, using a quality improvement tool to guide improvements in IPV protocols (e.g., responding to disclosures), staff training, support for staff related to secondary trauma, referral processes, and educational materials (e.g., posters, safety cards) (Miller-Walfish et al. 2021). Finally, one article described a systems-level demonstration project to improve IPV detection and response in community clinics by distributing a survey to facilitate IPV disclosure, conducting a social marketing campaign for health center staff, and having an on-site IPV advocate to distribute a survey, review results, and counsel/refer individuals at risk (Rhodes et al. 2014). The remaining three (42.9%) universal intervention studies evaluated Green Dot, a bystander-based prevention strategy that uses the power of peer social and cultural norms to reduce IPV, which was delivered as universal education to high school students (Coker et al. 2017, 2020; Edwards, Banyard, et al. 2022).

Selective interventions, focused on populations at elevated risk of IPV victimization and/or perpetration, comprised the largest category of studies (*n* = 29, 64.4%). Most of these studies (*n* = 24, 82.8%) examined screening and referral interventions, where individuals at risk of experiencing IPV (e.g., perinatal women) were screened with an assessment tool and then referred to community IPV resources and services. For example, the Behavioral Health Integrated Centralized Intake project introduced electronic behavioral health risk screening into the centralized intake process for home visiting agencies, and women who disclosed IPV were provided psychosocial support, information, and guided referrals to community resources (Price et al. 2017). Many (*n* = 23, 79.3%) of the interventions incorporated counseling and/or empowerment for populations at risk (e.g., to develop relationship and problem-solving skills, affirm strength, prompt self-reflection, and empower individuals to seek help), 22 (75.9%) studies described interventions in the context of home visiting programs, and 16 (55.2%) studies aimed to build IPV awareness or education. For example, the DOVE intervention covered all these within a home visiting intervention, where home visitors screened for IPV, leading discussions using a brochure about IPV health consequences, homicide risk factors, safety planning, and community resources (Bacchus et al. 2016). The Home Visitation Enhancing Linkages Project (HELP), also within a home visiting program, incorporated screening for IPV, connecting those who screened positive to appropriate providers, and then provided support to facilitate treatment retention and resolve any barriers to attendance (Dauber et al. 2017, 2019). Some studies incorporated culturally-tailored interventions, such as Sí, Yo Puedo, a Spanish educational program to raise awareness and provide IPV education, promote self-esteem, understand healthy relationships within a cultural framework, and empower women to access resources and support systems or Weaving Healthy Families, an intervention for Native American caregivers to promote positive parenting, emotional regulation, and resilience infused with Tribal content and modalities (Marrs Fuchsel 2014; McKinley and Theall 2021).

Finally, nine studies (20.0%) focused on an indicated intervention specifically for individuals involved in IPV victimization and/or perpetration to prevent further violence and mitigate harm of the violence that has already occurred. All nine (100.0%) interventions incorporated counseling and/or empowerment to develop relationship and problem-solving skills, affirm strengths, prompt self-reflection, and empower individuals to seek help (Carney and Buttell 2004; Hughes and Rasmussen 2010; Kramer et al. 2012; Marrs Fuchsel and Hysjulien 2013; McFarlane et al. 2006; McNamara et al. 2008; Rodgers et al. 2017). The interventions described in four of these studies (44.4%) also aimed to build IPV awareness or education, including a culturally-specific program for African American survivors with an Afrocentric curriculum (Gillum 2008), developing survivors’ interpersonal skills within a collaborative care model (Kramer et al. 2012), a culturally-competent psychoeducational curriculum for Spanish-speaking survivors to increase awareness and self-esteem (Marrs Fuchsel and Hysjulien 2013), and a nurse case management model incorporating safety planning and supportive listening (McFarlane et al. 2006). Lastly, four of the interventions (44.4%) involved facilitated or guided referrals to relevant community, social, and economic resources (Kramer et al. 2012; McFarlane et al. 2006; Parker et al. 1999; Rodgers et al. 2017).

### 3.4 | Evaluation of Outcomes

Most studies (*n* = 29, 64.4%) focused on intervention implementation, changes in IPV assessment or identification, program engagement or retention among the focus populations, changes in attitudes or knowledge, adoption of safety behaviors, or qualitative reflections on the intervention design or delivery (e.g., Eddy et al. 2008; Jack et al. 2023; Rodgers et al. 2017; Schanen et al. 2017). For example, the IMPACT intervention was associated with increased IPV-related knowledge and self-efficacy to use best practice IPV prevention strategies (Cox et al. 2010). Many of these studies described important potential mediators between the intervention and IPV behaviors, such as changes in IPV knowledge, identification of IPV, or referrals to services, but did not evaluate the interventions for their effects on or association with IPV behavioral outcomes (e.g., Dauber et al. 2019; Drabkin et al. 2021; Marrs Fuchsel 2014; Price et al. 2017; Shepard et al. 1999).

Among the 16 studies (35.6%) that evaluated whether the intervention was associated with IPV behavioral outcomes, 10 studies (62.5%) described positive program effects on IPV behaviors with the intervention reducing largely self-reported IPV victimization, and in some cases, perpetration (Bair-Merritt et al. 2010; Carney and Buttell 2004; Coker et al. 2017, 2020; Easterbrooks et al. 2021; Jacobs et al. 2016; Kiely et al. 2010; McFarlane et al. 2006; Parker et al. 1999; Sharps et al. 2016). These interventions with positive effects spanned education, homes, community clinics, and social service settings. For the studies examining the randomized controlled trial of the Augmented Nurse-Family Partnership home visiting program, several had null findings for the self-reported IPV victimization and perpetration outcomes (Feder et al. 2018; Jack et al. 2019; Li et al. 2022; Olds et al. 2004, 2010).

## 4 | Discussion

The results of this scoping review highlight the important role that community health professionals have in preventing and addressing IPV. We found numerous examples of the community-focused roles these professionals play in addressing upstream determinants of IPV and downstream impacts of IPV, with several programs reporting positive effects on IPV-related behaviors. The community health professionals described were most commonly nurses (i.e. registered nurses in roles as community health nurses or public health nurses), suggesting that they have, or are seen by researchers and public health leaders as potentially having particularly prominent roles in IPV prevention and response in communities (Bekemeier 1995; Hughes 2010).

The interventions identified in this review underscore the importance of cross-sector collaboration, which aligns with prior literature emphasizing the need for coordinated interventions across multiple sectors to reduce violence (Bacchus et al. 2024). This may be especially true for universal prevention activities in community settings; indeed, our identified examples of capacity building and school-based interventions necessarily involved several sectors and actors to coordinate delivery of education and services and to make effective referrals (Coker et al. 2017, 2020; Cox et al. 2010; Drabkin et al. 2021; Edwards, Banyard, et al. 2022; Miller-Walfish et al. 2021; Rhodes et al. 2014). This aligns with prior programs that emphasize collaboration among a multisectoral group of agencies to implement community-based prevention programs (Crooks et al. 2018). In addition, because IPV survivors can be identified and supported by multiple systems, interventions can facilitate “warm handoffs” and connection to critical social, economic, and community resources if multi-sector collaboration is working effectively.

Overall, we found a limited number of studies focused on broad, community-level primary prevention interventions, as much of the research we identified described intervening after IPV had occurred. This orientation towards response mirrors broader patterns of resource allocation towards healthcare, legal, and social service responses after violence has already taken place (Peterson et al. 2024; Wanni Arachchige Dona et al. 2025). Even for the universal interventions we identified, most were focused on capacity building (e.g., increasing knowledge, self-efficacy, protocols) for future interventions while a few universal interventions were focused on youth and in schools (Coker et al. 2017, 2020; Edwards, Banyard, et al. 2022). While early education is key, primary prevention located exclusively in schools misses opportunities to reach a broader population and to reinforce messages learned in school. In addition, school-based interventions and research often face barriers such as parental discomfort with the topic and concerns about traumatizing students, that can be challenging to overcome, particularly in communities more resistant to such efforts (Adhia et al. 2024; Edwards, Orchowski, et al. 2023). That we found relatively fewer universal interventions aligns with a recent review on community health worker core roles in violence prevention, which found that fewer than a third of programs included community-focused activities to address upstream and structural determinants of health inequities (Barbero et al. 2022). Given the scale of IPV’s societal costs, the relative scarcity of universal, community-level interventions also represents a missed opportunity to invest in primary prevention as a high-yield public health strategy with the potential to reduce downstream costs (Peterson et al. 2018; Sheppard et al. 2024).

Several studies highlighted the need for additional training for community health professionals to be able to effectively address IPV. Interprofessional education (e.g., between social work and nursing professionals) could be leveraged to provide training for addressing IPV, including enhancing comfort levels and knowledge of IPV (Childress et al. 2024). Such training is often focused on intervening in healthcare settings and with healthcare providers (Crombie et al. 2017; Hegarty et al. 2020), but future educational efforts should focus on expanding training on how to effectively collaborate across sectors and develop protocols to advance primary prevention in community settings. Nurses were the most frequently identified professionals in the interventions reviewed; thus, findings suggest that nurses in community settings are well-positioned to address IPV reports, safety planning, and referrals to resources. For nurses specifically, training could leverage the skills public health nurses already have in engaging communities and working across sectors (Kett et al. 2022, 2025), but apply these skills to the context of community-based IPV prevention. Since community health professionals have different practice scopes and skills depending on their profession (e.g., nurse vs. social worker), training should be tailored to the roles and functions of the different professions identified in this review. In all professions, emotional demands of this work underscore the importance of trauma-informed training, supervision, and organizational supports to mitigate risks of vicarious trauma and burnout (Lundy and Crawford 2024).

The number of types of community health professionals involved and the range of interventions studies conducted was rather limited. For example, approximately half of studies were focused on nurses and on home visitors, many of those related to the Nurse-Family Partnership program. Much of this focus may have been related to what types of research funding was available and obtained and the related data available to researchers, including home visiting-related research specific to Nurse-Family Partnership (Condon 2019). This pattern suggests that funding priorities play an important role in shaping the evidence base, potentially focusing on individual-level interventions over community-level interventions which may be harder to evaluate, particularly in the short-term. This investment in home visiting programs also emphasizes the importance of the perinatal period for addressing IPV, perhaps due to increased risk during this period (Wong et al. 2023).

Regarding public health nurses specifically, we found very little IPV-related research specific to these nurses, aside from home visiting program studies, despite research that has shown public health nurses to be particularly skilled in relationship-building, community advocacy, building a broad knowledge base, and advancing health equity (Kett et al. 2022, 2025). While this prior research by Kett and colleagues was not focused on IPV, some of these same strengths are applicable to IPV-related prevention activities that which occur outside of home visiting. This may be an important area of future research and for expanded, community-level IPV prevention training, given that public health nurses appear particularly equipped to engage in community-based prevention (Dickson & Tutty 1996; Hughes 2010).

Finally, the studies included in this review underline the need for and importance of culturally tailored, appropriate, and responsive interventions (Bagwell-Gray et al. 2021; Henriksen et al. 2023). We identified several examples of programs that incorporated modalities and content aligned with the culture and norms of their participant populations, including Sí, Yo Puedo for Spanish-speaking women (Marrs Fuchsel 2014), Weaving Healthy Families for Native American caregivers (McKinley and Theall 2021), and an Afrocentric curriculum for African American IPV survivors (Gillum 2008). These programs were reported by survivors as beneficial in meeting their needs by incorporating relevant cultural values, norms, and attitudes. To bridge the persistent research-to-practice gap, such tailored interventions may be well-suited for implementation science approaches, including effectiveness-implementation hybrid designs, use of established frameworks (e.g., the Consolidated Framework for Implementation Research or the RE-AIM framework), or community-engaged implementation mapping to develop strategies that support implementation of evidence-based interventions in real-world settings (Fernandez et al. 2019; Miller et al. 2021; Sabri et al. 2026). These approaches could generate practice-relevant evidence to guide community health professionals in assuring effective IPV interventions are delivered for and with the unique communities they serve.

### 4.1 | Limitations

Results from this scoping review should be interpreted in the context of some limitations. First, our included studies were highly heterogeneous, prohibiting any meta-analysis or conclusions about certain types of interventions. Nearly two thirds of the studies that evaluated whether the intervention was associated with IPV behavioral outcomes reported positive program effects, but there may be publication bias where interventions that had no significant effects were not published. The included studies also measured IPV in different ways (e.g., physical only, physical and sexual; past-year, lifetime) in different settings. We also only included studies that were based in the U.S. and published in English and did not search the grey literature. Examining program reports and evaluations not available in peer-reviewed literature would likely provide additional interventions and activities that community health professionals could engage in to address IPV. While we incorporated several specific search terms to capture a range of community health professionals, we may not have captured every role given the breadth and variation in titles (e.g., all types of licensed mental health counselors). Moreover, given our focus on interventions delivered by community health professionals, we did not comprehensively capture structural interventions for IPV (e.g., laws and policies) (Bourey et al. 2015; Clark et al. 2022; Sabri et al. 2023).

## 5 | Conclusion

IPV remains a serious and complex public health issue requiring coordinated, community-based prevention and response. This scoping review identifies several types of interventions that illustrate the potential for community health professionals to effectively address IPV, largely through screening, referral, and empowerment-based approaches. The predominance of selective and indicated interventions underscores the need for universal, community-level primary prevention efforts, with more research needed to guide implementation of such efforts. These findings also indicate a need for expanded workforce training, organizational support, and funding to promote and sustain prevention work by community health professionals.

## Supplementary Material

Supplementary

Additional supporting information can be found online in the Supporting Information section.

**Supporting File 1:** phn70166-supp-0001-SuppMat.docx

## Figures and Tables

**Table 1.** Peer-reviewed studies related to intimate partner violence (IPV) prevention by community health professionals

Study characteristic	No. of studies (%) n=45
Publication year	
Before 2000	2 (4.4)
2000–2009	8 (17.8)
2010–2019	24 (53.3)
2020 and after	11 (24.4)
Study design	
Quantitative only	29 (64.4)
Qualitative only	10 (22.2)
Quantitative and qualitative	6 (13.3)
Community setting^±^	
Homes	24 (53.3)
Community clinics (e.g., health, Women, Infants, Children)	12 (26.7)
Social service (e.g., shelters, community-based organizations)	7 (15.6)
Education	5 (11.1)
Justice/legal	4 (8.9)
Other (e.g., online, other community venues)	4 (8.9)
Community health professional role^±^	
Nurse (e.g., registered nurse practicing as public health nurse or community health nurse)	22 (48.9)
Home visitor	21 (46.7)
IPV organization staff (e.g., advocate)	12 (26.7)
Community health worker (e.g., paraprofessionals, health educators)	11 (24.4)
Social worker	7 (15.6)
Intervention population	
Perinatal women	24 (53.3)
IPV survivors	8 (17.8)
At-risk individuals	5 (11.1)
Students	4 (8.9)
Community health professionals	3 (6.7)
IPV perpetrators	1 (2.2)
Evaluation for IPV outcomes	
Yes	16 (35.6)
No	29 (64.4)

± Studies could be included in multiple categories, so percentages may total greater than 100%

**Table 2.** Intervention types by prevention classification

	No. of studies (%) n=45
**Universal (entire population group regardless of risk)**	**7 (15.6)**
Organizational capacity building	4 (57.1)
Bystander training	3 (42.9)
**Selective (elevated risk populations)**	**29 (64.4)**
Screening/referral	24 (82.8)
Empowerment/counseling program	23 (79.3)
Home visiting	22 (75.9)
IPV awareness/education	16 (55.2)
**Indicated (IPV-involved individuals)**	**9 (20.0)**
Empowerment/counseling program	9 (100.0)
IPV awareness/education	4 (44.4)
Referral	4 (44.4)

Note: Percentages for sub-types of interventions are out of the total parent category (e.g., universal interventions are the parent category for organizational capacity building and bystander training). In addition, interventions can be categorized into multiple sub-types (e.g., an intervention can be counted in screening/referral and home visiting), so percentages within categories add to >100%.

## Data Availability

The data that support the findings of this study are available from the corresponding author upon reasonable request.
