# Supplementary material for: The Role of Community Health Professionals in Addressing Intimate Partner Violence: A Scoping Review
Source: Public Health Nurs. 2026 Aug 4;43(6):1634–43. doi: 10.1111/phn.70166 (PMC13580420; doi:10.1111/phn.70166)
Supplement: Supplementary file 1 [file PHN-43-1634-s001.docx]

**Supplemental Table 1**. Search terms used to identify relevant publications related to community health professionals and intimate partner violence

| **Database** | **Search terms** |
| --- | --- |
| **PubMed**  (*n* = 1,944) | ("House Calls"[Mesh] OR "Community Health Workers"[Mesh] OR "Social Workers"[Mesh] OR "Health Educators"[Mesh] OR "Doulas"[Mesh] OR "Midwifery"[Mesh] OR "Nurses, Public Health"[Mesh] OR "home-visiting nurse*" OR "community health work*" OR "social work*" OR "community psychologist*" OR "health educator*" OR "public health nurse*" OR "community health nurse*" OR "health visitors" OR doulas OR midwi* OR "peer-based support" OR "community advocate" OR "outreach worker" OR "community health aides" OR "community aides" OR "health aides") AND ("Intimate Partner Violence"[Mesh] OR "Domestic Violence"[Mesh] OR "domestic violence" OR "domestic abuse" OR "intimate partner violence" OR "family violence" OR "partner abuse" OR "partner violence") NOT ("Child Abuse"[Mesh] OR "child abuse") AND ("United States"[Mesh] OR “United States” OR American OR “USA” OR “U.S.A” OR “U.S.” OR Appalach* OR Atlantic OR “Great Lakes” OR “Mid-Atlantic” OR Midwest OR Midwestern OR “New England” OR Northwestern OR “Pacific Northwest” OR “Pacific States” OR Southeastern OR Southwest OR Southern OR Alabama OR Alaska OR Arizona OR Arkansas OR California OR Colorado OR Connecticut OR Delaware OR “DC” OR “District of Columbia” OR Florida OR Georgia OR Hawaii OR Idaho OR Illinois OR Indiana OR Iowa OR Kansas OR Kentucky OR Louisiana OR Maine OR Maryland OR Massachusetts OR Michigan OR Minnesota OR Mississippi OR Missouri OR Montana OR Nebraska OR Nevada OR “New Hampshire” OR “New Jersey” OR “New Mexico” OR “New York” OR “North Carolina” OR “North Dakota” OR Ohio OR Oklahoma OR Oregon OR Pennsylvania OR “Rhode Island” OR South Carolina OR “South Dakota” OR Tennessee OR Texas OR Utah OR Vermont OR Virginia OR Washington OR West Virginia OR Wisconsin OR Wyoming OR Anchorage OR “Ann Arbor” OR Atlanta OR Baltimore OR “Baton Rouge” OR Berkeley OR Bethesda OR Birmingham OR Boise OR Boston OR Bozeman OR Burlington OR Chicago OR Cincinnati OR Cleveland OR Columbus OR Denver OR Detroit OR Fairbanks OR Farmington OR Flagstaff OR Gainesville OR Hartford OR Honolulu OR Houston OR Huntsville OR Indianapolis OR Jackson OR Jacksonville OR “Las Vegas” OR Lexington OR Lincoln OR “Little Rock” OR “Los Angeles” OR Louisville OR Memphis OR Miami OR Minneapolis OR Missoula OR Montgomery OR Nashville OR “New Orleans” OR Newark OR Omaha OR Philadelphia OR Phoenix OR Portland OR Providence OR Richmond OR Rochester OR Rockville OR “Saint Paul” OR “San Diego” OR “San Francisco” OR “St Paul” OR Seattle OR Shreveport OR Stanford OR Tallahassee OR Tampa OR Tucson OR Urbana OR Wichita OR Wilmington) |
| **CINAHL**  (*n* = 850) | (MH "Home Visits" OR MH "Community Health Workers" OR MH "Community Health Nurses+" OR MH "Social Workers" OR MH "Health Educators+" OR MH "Midwives+" OR MH "Public Health Nurses" OR MH "Public Health Nursing" OR "home-visiting nurse*" OR "community health work*" OR "social work*" OR "community psychologist*" OR "health educator*" OR "public health nurse*" OR "community health nurse*" OR "health visitors" OR doulas OR midwi* OR "peer-based support" OR "community advocate" OR "outreach worker" OR "community health aides" OR "community aides" OR "health aides") AND (MH "Intimate Partner Violence" OR MH "Domestic Violence+" OR "domestic violence" OR "domestic abuse" OR "intimate partner violence" OR "family violence" OR "partner abuse" OR "partner violence") NOT (MH "Child Abuse+" OR "child abuse")  *Filters: Geography: USA* |
| **Embase**  (*n* = 812) | ('home visit'/exp OR 'health auxiliary'/exp OR 'social worker'/exp OR 'health educator'/exp OR 'doula'/exp OR 'midwife'/exp OR 'home-visiting nurse' OR 'community health work' OR 'social work' OR 'community psychologist' OR 'health educator' OR 'public health nurse' OR 'community health nurse' OR 'health visitors' OR doulas OR midwi* OR 'peer-based support' OR 'community advocate' OR 'outreach worker' OR 'community health aides' OR 'community aides' OR 'health aides') AND ('partner violence'/exp OR 'domestic violence'/exp OR 'domestic violence' OR 'domestic abuse' OR 'intimate partner violence' OR 'family violence' OR 'partner abuse' OR 'partner violence') NOT ('child abuse'/exp OR 'child abuse') AND [embase]/lim AND ('United States'/exp OR 'American'/exp OR ‘United States’ OR American OR ‘USA’ OR ‘U.S.A’ OR ‘U.S.’ OR Appalach* OR Atlantic OR ‘Great Lakes’ OR ‘Mid-Atlantic’ OR Midwest OR Midwestern OR ‘New England’ OR Northwestern OR ‘Pacific Northwest’ OR ‘Pacific States’ OR Southeastern OR Southwest OR Southern OR Alabama OR Alaska OR Arizona OR Arkansas OR California OR Colorado OR Connecticut OR Delaware OR ‘DC’ OR ‘District of Columbia’ OR Florida OR Georgia OR Hawaii OR Idaho OR Illinois OR Indiana OR Iowa OR Kansas OR Kentucky OR Louisiana OR Maine OR Maryland OR Massachusetts OR Michigan OR Minnesota OR Mississippi OR Missouri OR Montana OR Nebraska OR Nevada OR ‘New Hampshire’ OR ‘New Jersey’ OR ‘New Mexico’ OR ‘New York’ OR ‘North Carolina’ OR ‘North Dakota’ OR Ohio OR Oklahoma OR Oregon OR Pennsylvania OR ‘Rhode Island’ OR South Carolina OR ‘South Dakota’ OR Tennessee OR Texas OR Utah OR Vermont OR Virginia OR Washington OR West Virginia OR Wisconsin OR Wyoming OR Anchorage OR ‘Ann Arbor’ OR Atlanta OR Baltimore OR ‘Baton Rouge’ OR Berkeley OR Bethesda OR Birmingham OR Boise OR Boston OR Bozeman OR Burlington OR Chicago OR Cincinnati OR Cleveland OR Columbus OR Denver OR Detroit OR Fairbanks OR Farmington OR Flagstaff OR Gainesville OR Hartford OR Honolulu OR Houston OR Huntsville OR Indianapolis OR Jackson OR Jacksonville OR ‘Las Vegas’ OR Lexington OR Lincoln OR ‘Little Rock’ OR ‘Los Angeles’ OR Louisville OR Memphis OR Miami OR Minneapolis OR Missoula OR Montgomery OR Nashville OR ‘New Orleans’ OR Newark OR Omaha OR Philadelphia OR Phoenix OR Portland OR Providence OR Richmond OR Rochester OR Rockville OR ‘Saint Paul’ OR ‘San Diego’ OR ‘San Francisco’ OR ‘St Paul’ OR Seattle OR Shreveport OR Stanford OR Tallahassee OR Tampa OR Tucson OR Urbana OR Wichita OR Wilmington)  *Filters: Sources: Embase* |
| **Web of Science**  (*n* = 408) | ("home-visiting nurse" OR "community health worker" OR "social work" OR “social worker” OR "community psychologist" OR "health educator" OR "public health nurse" OR "community health nurse" OR "health visitors" OR doulas OR midwi* OR "peer-based support" OR "community advocate" OR "outreach worker" OR "community health aides" OR "community aides" OR "health aides") AND ("domestic violence" OR "domestic abuse" OR "intimate partner violence" OR "family violence" OR "partner abuse" OR "partner violence") NOT ("child abuse")  *Filters: Countries/Regions: United States* |

**Supplemental Table 2**. Characteristics of studies related to intimate partner violence (IPV) prevention by community health professionals

| **Reference** | **Setting/sector** | **Community health professional** | **Focus population** | **Types of IPV addressed** | **Study design** | **Intervention description** | **Evaluation of IPV behaviors** | **Major IPV findings** |
| --- | --- | --- | --- | --- | --- | --- | --- | --- |
| **Universal** |  |  |  |  |  |  |  |  |
| Coker et al. (2017) | Education | IPV organization staff (rape crisis center educators) | High school students | Physical, emotional | Quantitative, RCT | *Green Dot*: schoolwide presentations on being engaged bystanders and recruitment of student popular opinion leaders to receive bystander training | Yes | Significantly fewer IPV victimization and perpetration events observed in intervention vs. control schools |
| Coker et al. (2020) | Education | IPV organization staff (rape crisis center educators) | High school students | Physical, emotional | Quantitative, RCT | *Green Dot*: schoolwide presentations on being engaged bystanders and recruitment of student popular opinion leaders to receive bystander training | Yes | Sexual majority, but not sexual minority, students experienced significant reductions in physical IPV victimization and perpetration |
| Cox et al. (2010) | Social service, community clinics, justice/legal | IPV organization staff (coordinated community response coalitions (CCRs)) | CCRs | General IPV | Quantitative, pre-post | *Domestic Violence Prevention Enhancements and Leadership Through Alliance (DELTA)*: CDC program to focus on primary prevention of IPV by funding state coalitions to engage in statewide primary prevention efforts and to provide training, technical assistance, and financial support to local communities | No | CCRs strengthened organizational structures (e.g., bylaws, goals), implemented organizational processes (e.g., mechanisms for decision-making), increased funding, and increased primary prevention activities |
| Drabkin et al. (2021) | Social service, community clinics, justice/legal, education, online | IPV organization staff (prevention professionals in counseling, legal work, medical care, shelter work, and schools) | IPV prevention professionals involved in direct prevention work | General IPV | Quantitative, RCT | *IMPACT:* 13 multimedia, online training modules designed to increase the capacity of IPV prevention professionals to effectively implement evidence-based IPV prevention programs | No | Compared to control participants, IMPACT participants significantly increased IPV-related knowledge and self-efficacy to utilize best practice IPV prevention strategies |
| Edwards et al. (2022) | Education, online | IPV organization staff | High school students | General dating violence | Quantitative, cross-sectional | *Green Dot*: schoolwide presentations on being engaged bystanders and recruitment of student opinion leaders to receive bystander training; other various town prevention activities (e.g., elective class about gender) | No | Active exposure and active participation were related to greater perceptions of collective efficacy and perceptions of social norms more intolerant of IPV |
|  |  |  |  |  |  |  |  |  |
| Miller-Walfish et al. (2021) | Community clinics | IPV organization staff (advocates), community health workers (safety net health care providers) | Health sites | General IPV | Quantitative, pre-post | *Domestic Violence and Health Care Partnerships (DVHCP):* promote collaboration between IPV advocates and health care sites; clinic lead used a Quality Assessment/Quality Improvement (QA/QI) tool to guide improvements in IPV protocols (e.g., responding to disclosures), staff training, support for staff related to secondary trauma, referral processes, and educational materials/resources (e.g., posters, safety cards) | No | Implementation of QA/QI tool was feasible with largest changes seen in: having written protocols for assessing IPV, providers distributing educational safety cards to patients, scripts for providers on how to assess and support survivors of IPV, trainings led by IPV agency advocates, and support for staff to discuss difficult cases |
| Rhodes et al. (2013) | Community clinics | IPV organization staff (advocates), social workers | Patients aged 18-64 years receiving care at community health clinic | Physical, sexual, emotional | Quantitative, pre-post; community-based participatory research | Demonstration project to improve IPV detection and response with 3 components: 1) distribution of the Social Health Survey to facilitate patient disclosure of IPV, 2) social marketing campaign for health center staff, 3) presence of on-site IPV advocate to distribute survey, review results, and counsel/refer individuals at risk | No | Increased number of IPV cases identified from baseline but could not sustain screening in routine care so detection reverted to baseline levels once the advocate was no longer present |
| **Selective** |  |  |  |  |  |  |  |  |
| Bacchus et al. (2016) | Homes | Home visitors (nurses, community health workers) | Perinatal women | Physical, sexual, emotional | Qualitative, interviews | *Domestic Violence Enhanced Home Visitation (DOVE):* empowerment intervention that discusses the DOVE brochure with information on IPV health consequences, risk assessment, safety planning, and community resources. Screening and intervention were done via home visitor or computer tablet | No | Women valued the opportunity to discuss IPV and access support; developing trust and safety planning were important features with need for need for post-IPV support services |
| Bacchus et al. (2016) | Homes | Home visitors (nurses, community health workers) | Perinatal women | Physical, sexual, emotional | Qualitative, interviews | *Domestic Violence Enhanced Home Visitation (DOVE):* empowerment intervention that discusses the DOVE brochure with information on IPV health consequences, risk assessment, safety planning, and community resources. Screening and intervention were done via home visitor or computer tablet | No | mHealth approach (i.e., computer tablet) can be used for safe IPV disclosure and to initiate IPV discussions, but training is needed to ensure home visitors can use the tablet to complement the therapeutic relationship |
| Bair-Merritt, et al. (2010) | Homes | Home visitors (paraprofessionals) | Families with infants at high risk for maltreatment | Physical, sexual, emotional | Quantitative, RCT | *Hawaii Healthy Start Program (HSP):* early-childhood home visitation from infants’ birth to three years that included linking families to community services and providing direct services (e.g., teaching about child development, role-modeling positive parenting and problem-solving strategies, offering emotional support) | Yes | Intervention participation was associated with significantly reduced maternal IPV victimization and perpetration (specifically physical violence) |
| Burnett et al. (2021) | Homes | Home visitors (nurses, community health workers) | Perinatal women | Physical, sexual, emotional | Qualitative, interviews | *Domestic Violence Enhanced Home Visitation (DOVE):* empowerment intervention that discusses the DOVE brochure with information on IPV health consequences, risk assessment, safety planning, and community resources. Screening and intervention were done via home visitor or computer tablet | No | Screening facilitated conversations about IPV. DOVE enhanced home visitors’ IPV knowledge and intervening capabilities. Establishing relationships before screening and providing training/support for delivery was helpful |
| Dauber et al. (2017) | Homes | Home visitors | Perinatal women at risk of child maltreatment | Physical, sexual, emotional | Quantitative, cross-sectional; qualitative, interviews | *Home Visitation Enhancing Linkages Project (HELP):* three-phase intervention – 1) Identify: screening for IPV, depression, substance use, 2) Connect: link those screening positive to treatment with motivational interviewing and case management, 3) Support: maintenance phase to facilitate retention, resolve attendance barriers, and provide support | No | Nearly all participants were screened at baseline; among positively screened clients, less than half received a referral or case management, and about half received a support intervention. Client disclosure of risk, barriers to treatment access, and systems integration affected HELP implementation |
| Dauber et al. (2019) | Homes | Home visitors | Perinatal women at risk of child maltreatment | Physical, sexual, emotional | Quantitative, quasi-experimental | *Home Visitation Enhancing Linkages Project (HELP):* three-phase intervention – 1) Identify: screening for IPV, depression, substance use, 2) Connect: link those screening positive to treatment with motivational interviewing and case management, 3) Support: maintenance phase to facilitate retention, resolve attendance barriers, and provide support | No | HELP significantly improved discussion of client risk of IPV during home visits, but had no impact on risk identification and minimal impact on referral |
| Davidov et al. (2021) | Homes | Home visitors (nurses, paraprofessionals) | Perinatal women | Physical, sexual, emotional | Qualitative, focus groups | Home visitors within *Healthy Families America, Maternal Infant Health Outreach Worker, Parents as Teachers*, and *Early Head Start* program models: strengthening screening and assessment, referrals to IPV resources and services, and developing safety plans with IPV-exposed women in rural, underserved areas | No | Barriers included nature of assessment tools, service unavailability in rural areas, and lack of training on safety planning. Facilitators included building trust and rapport, providing anticipatory guidance for referrals, and tailoring safety plans |
| Easterbrooks et al. (2021) | Homes | Home visitors (paraprofessionals) | Perinatal women (first-time parents under age 21) | Physical, sexual, emotional | Quantitative, RCT | *Healthy Families Massachusetts*: statewide, voluntary newborn home visitation program providing home visits, goal-setting activities, group-based activities, and referral service from infants’ birth to three years | Yes | Significant home visiting program effects on maternal well-being (i.e., parenting distress and risky behaviors) were mediated by reductions in IPV |
| Eddy et al. (2008) | Homes | Home visitors (nurses, social workers, paraprofessionals) | Pregnant women | Physical, sexual, emotional | Quantitative, cross-sectional; qualitative, focus groups | *Domestic Violence Enhanced Home Visitation (DOVE):* empowerment intervention that discusses the DOVE brochure with information on IPV health consequences, risk assessment, safety planning, and community resources | No | Barriers included home visitor stress and discomfort initiating IPV conversations. Facilitators included prior experience with IPV disclosure, building rapport, and using voice inflection/non-verbal cues |
| Feder et al. (2018) | Homes | Home visitors (community health nurses) | Pregnant women (low-income, first-time) | Physical, sexual, emotional | Quantitative, RCT | *Augmented Nurse Family Partnership (NFP+)*: home visits from the second trimester to the child's 2^nd^ birthday with discussion about effective parenting and a positive life course augmented with IPV screening. If IPV was disclosed, nurses delivered a brochure-based intervention with safety planning/referrals and skills-based relationship curriculum (*Within My Reach*) | Yes | No significant main effects of NFP for any of the IPV victimization or perpetration outcomes; however, NFP significantly reduced physical and IPV victimization and psychological IPV perpetration among women not experiencing IPV at baseline. NFP was ineffective or potentially harmful for those already experiencing IPV at baseline |
| Godecker et al. (2013) | Community clinics | Nurses (registered), community health workers | Pregnant women | Physical, sexual, emotional | Quantitative, cross-sectional | *Prenatal Risk Overview (PRO)*: standardized psychosocial risk screening interview administered at intake to prenatal women that addresses 13 domains including IPV and classifies into high vs. moderate risk categories | No | Community health workers were found to be able to effectively conduct risk screenings and elicited more self-reported risk for some domains than nurses, especially related to basic needs (IPV was similar) |
| Jack et al. (2017) | Homes | Home visitors (registered nurses) | Pregnant women (low-income, first-time) | Physical, sexual, emotional | Qualitative, interviews, focus groups | *Nurse Family Partnership (NFP)*: home visits from the second trimester to the child’s 2^nd^ birthday discussing content on personal health, environmental health, friends and family, maternal role, use of healthcare and human services, and life course development | No | Women were more likely to disclose IPV when nurses initiated nonstructured discussions about parenting, safety, or relationships than when nurses used structured screening tools. Nurses need knowledge and skills to tailor approaches |
| Jack et al. (2019) | Homes | Home visitors (registered nurses) | Pregnant women (low-income, first-time) | Physical, sexual, emotional | Quantitative, RCT | *Augmented Nurse Family Partnership (NFP+)*: home visits from the second trimester to the child's 2^nd^ birthday with discussion about effective parenting and a positive life course augmented with IPV screening. If IPV was disclosed, nurses delivered a brochure-based intervention with safety planning/referrals and skills-based relationship curriculum (*Within My Reach*) | Yes | NFP+ showed non-significant improvements in IPV victimization and quality of life |
| Jack et al. (2023) | Homes | Home visitors (registered nurses) | Pregnant women (low-income, first-time) | Physical, sexual, emotional | Qualitative, interviews and focus groups | *Augmented Nurse Family Partnership (NFP+)*: home visits from the second trimester to the child's 2^nd^ birthday with discussion about effective parenting and a positive life course augmented with IPV screening. If IPV was disclosed, nurses delivered a brochure-based intervention with safety planning/referrals and skills-based relationship curriculum (*Within My Reach*) | No | Integration of IPV intervention into NFP was negatively impacted by lack of centralized program support and competing program demands. Other factors influencing ability to address IPV included supervisor capacity, preservation of nurse-client relationship, lack of privacy in home visits, and lack of experience/preparedness |
| Jacobs et al. (2016) | Homes | Home visitors (paraprofessionals) | Perinatal women (first-time parents between 16-20 years old) | Physical, sexual, emotional | Quantitative, RCT | *Healthy Families Massachusetts (HFM)*: statewide, voluntary newborn home visitation program providing home visits, goal-setting activities, group-based activities, and referral service from infants’ birth to three years | Yes | HFM significantly reduced IPV perpetration in addition having positive influences on parenting stress, condom use, risky behaviors, and college attendance |
| Kelly et al. (2007) | Community venues (e.g., driveways, patios, meeting rooms) | Community health workers (promotoras) | At-risk women (low-income, Spanish-speaking) | Physical, sexual | Qualitative, observation, field notes, journals, open-ended surveys | *Promotoras de Buena Vida*: trains women to be promotoras through 18-hour program discussing violence against women, practicing presentation skills, and providing critiques of each other’s work. Training covered dynamics and impacts of violence, myths, reasons women don't leave, community resources, and strategies for sharing what they've learned. After the training, the women organized 80 different community presentations to share learnings with other women | No | Promotoras gained knowledge of IPV and its personal impact and developed skills and sense of themselves as community benefactors. Women who attended community presentations found the information personally applicable, wanted to spread learnings in the community, and wanted to include men |
| Kiely et al. (2010) | Community clinics | Social workers, community health workers (psychologists) | Perinatal women | Physical, sexual | Quantitative, RCT | Cognitive behavioral individualized intervention (minimum of 4 sessions) based on empowerment theory providing information about types of abuse, cycle of violence, Danger Assessment to assess risks, preventive options, safety planning, and community resources | Yes | Women randomized to intervention were significantly less likely to have recurrent IPV victimization, particularly women who experienced minor IPV and physical IPV |
| Li et al. (2022) | Homes | Home visitors (nurses) | Pregnant women (low-income, first-time) | Physical, sexual, emotional | Quantitative, RCT | *Augmented Nurse Family Partnership (NFP+)*: home visits from the second trimester to the child's 2^nd^ birthday with discussion about effective parenting and a positive life course augmented with IPV screening. If IPV was disclosed, nurses delivered a brochure-based intervention with safety planning/referrals and skills-based relationship curriculum (*Within My Reach*) | Yes | NFP+ was not effective for Latinx mothers by nativity, and the nativity gap diminished over time: US-born Latinx mothers reported IPV at similar levels during the program, while foreign-born Latinx mothers reported higher levels of IPV after the program likely due to better detection /reporting of IPV |
| MarrsFuchsel (2014) | Community clinics | Social workers | Immigrant Latina women | General IPV | Qualitative, focus groups | *Sí, Yo Puedo (formerly Domestic Violence Intervention Model)*: culturally-competent educational program conducted in Spanish to provide IPV education, promote self-esteem, understand healthy relationships within a cultural framework, and empower women to access resources and support systems | No | Women reported improvement in self-esteem, knowledge of IPV, and effective communication with partners, and experienced the process of empowerment through participation |
| McKinley & Theall (2021) | Community venues (church sites) | Community health workers (trusted, usually Native American, community members) | Native American caregivers with at least one child aged 12+ | Physical, sexual, emotional | Quantitative, pre-post | *Weaving Healthy Families (WHF)*: family-focused, culturally-tailored intervention to promote positive parenting, emotional regulation, and resilience, with Tribal content (e.g., talking circle, Tribal foodways, gender/colonization) infused throughout | No | Pilot results showed reduced IPV and increased healthy conflict resolution skills in addition to improvements in substance use, depressive symptoms, family resilience, emotional regulation, social support, and adolescent health-related quality of life |
| Olds et al. (2004) | Homes | Home visitors (nurses) | Perinatal women | Physical | Quantitative, RCT | Nurse home-visiting services during pregnancy until child's 2^nd^ birthday with the goals of promoting women's healthy prenatal behaviors, promoting competent care of children, and encouraging parents to enhance stability. Nurses helped families make use of health and human services and attempted to involve other family and friends | Yes | No statistically significant program effects on women’s experiences of IPV, but women in the program had significantly longer relationships with current partners |
| Olds et al. (2004) | Homes | Home visitors (nurses, paraprofessionals) | Perinatal women | Physical | Quantitative, RCT | Nurse home-visiting services during pregnancy until child's 2^nd^ birthday with the goals of promoting women's healthy prenatal behaviors, promoting competent care of children, and encouraging parents to enhance stability. Nurses helped families make use of health and human services and attempted to involve other family and friends | Yes | No statistically significant paraprofessional effects on on women’s experiences of IPV, but nurse-visited women who were married or cohabiting experienced significantly less IPV in the 2 years after the end of the program |
| Olds et al. (2010) | Homes | Home visitors (nurses) | Perinatal women | Physical | Quantitative, RCT | Nurse home-visiting services during pregnancy until child's 2^nd^ birthday with the goals of promoting women's healthy prenatal behaviors, promoting competent care of children, and encouraging parents to enhance stability. Nurses helped families make use of health and human services and attempted to involve other family and friends | Yes | No statistically significant program effects on women’s experiences of IPV through age 12 of their child |
| Price et al. (2017) | Homes | Community health workers (paraprofessionals) | Perinatal women | Physical, emotional | Quantitative, cross-sectional, qualitative, interviews | *Behavioral Health Integrated Centralized Intake (BH-CI)* project: introduced electronic behavioral health risk screening into the centralized intake process for each home visiting agency. Staff were trained in motivational interviewing. Women who disclosed IPV were provided psychosocial support, information, and guided referrals to community resources, and a coordinator followed up to ensure communication from referrals | No | Among women who endorsed historical IPV experiences, most were referred to home visiting programs with others referred to substance use, mental health, violence prevention, or other community programs /resources |
| Schanen et al. (2017) | Education | Nurses (public health), community health workers (community health educators) | Native American high school students | General IPV | Quantitative, pre-post | *Discovery Dating*: 14 session curriculum using several approaches (e.g., interactive activities, reflection, repetition of skills, celebration) to address 8 components including goal setting, examining benefits and pitfalls in relationships, impacts of decision making, mentoring, and managing life chaos | No | The curriculum was associated with significant increases in students’ personal agency, while resilience and self-efficacy were not significantly improved |
| Sharps et al. (2013) | Homes | Home visitors (nurses) | Perinatal women | Physical, sexual, emotional | Quantitative, RCT | *Domestic Violence Enhanced Home Visitation (DOVE):* empowerment intervention that discusses the DOVE brochure with information on IPV health consequences, risk assessment, safety planning, and community resources. Screening and intervention were done via home visitor or computer tablet | No | Retention rates from baseline for women who received the DOVE intervention decreased from delivery to 12 months with no differences in retention rates for women participating in the NFP program vs. DOVE |
| Sharps et al. (2016) | Homes | Home visitors (community health nurses) | Perinatal women | Physical, sexual, emotional | Quantitative, RCT | *Domestic Violence Enhanced Home Visitation (DOVE):* empowerment intervention that discusses the DOVE brochure with information on IPV health consequences, risk assessment, safety planning, and community resources | Yes | Significant decrease in IPV over time for all women with a larger and statistically significant decrease for women receiving DOVE vs. standard home visiting |
| Sharps et al. (2024) | Homes | Home visitors (nurses, social workers, paraprofessionals) | Pregnant women | Physical, sexual, emotional | Quantitative, RCT | *Domestic Violence Enhanced Home Visitation (DOVE):* empowerment intervention that discusses the DOVE brochure with information on IPV health consequences, risk assessment, safety planning, and community resources. Screening and intervention were done via paper-pencil or computer tablet | No | No significant differences in IPV prevalence between paper-pencil vs. tablet screening methods |
| Shepard et al. (1999) | Homes | Home visitors (public health nurses) | Perinatal women | Physical, emotional | Quantitative, pre-post | *Enhanced Domestic Abuse Intervention Project*: enhanced IPV assessment protocol for use in home visits to increase the identification and referral rates of women experiencing IPV | No | Protocol resulted in non-significant increases in IPV identification; significantly more women were provided with IPV resources and referred to services |
| **Indicated** |  |  |  |  |  |  |  |  |
| Carney & Buttell (2004) | Social service, justice/legal | IPV organization staff (counselors) | Women perpetrators court-ordered to treatment | Physical, sexual, emotional | Quantitative, pre-post | *Batterer Intervention Program:* structured 16-week group treatment program  that focuses on anger management and skills  development, incorporating confrontation, therapy, and educational components | Yes | Women who completed treatment were significantly less passive/aggressive and less likely to use physical force on their partner |
| Gillum (2008) | Social service | IPV organization staff (advocates) | African American female IPV survivors | General IPV | Qualitative, interviews | Culturally specific IPV program delivered by African American staff involving Afrocentric curriculum using a family-centered, spiritually-based, and holistic approach including support groups, mental health services, and substance abuse treatment | No | Culturally specific services were beneficial to survivors in meeting their needs through incorporating relevant cultural values, norms, and attitudes |
| Hughes & Rasmussen (2010) | Social service | IPV organization staff (counselors) | IPV survivors seeking services from shelter | Physical, sexual, emotional, stalking | Quantitative, pre-post; qualitative, interviews | *Motivational Interviewing:* reflects and amplifies feelings, affirms strengths and resilience, and reframes negative perceptions using exploratory questions and problem-solving methods to prompt survivor’s self-reflection and examination of the situation | No | Survivors receiving motivational interviewing focused on their strengths (e.g., exhibited self-determination, and self-esteem) and had a clearer understanding of how their partners used power and control |
| Kramer et al. (2012) | Community clinics, homes, social service, justice/legal | Nurses (clinical nurse specialist, registered nurse case manager), IPV organization staff | Pregnant and postpartum IPV survivors | Physical, sexual, emotional | Quantitative, pre-post, qualitative, interviews | *Safe Mom, Safe Baby (SMSB):* combines registered nurse case management, advocacy services of a community-based IPV agency, and perinatal care to empower survivors to engage with healthcare providers and other services (e.g., crisis intervention, emotional support, advocacy) and adopt safety behaviors | No | Women receiving SMSB reported high satisfaction with the program, increased numbers of implemented safety behaviors, and increased readiness to initiate significant life changes |
| MarrsFuchsel & Hysjulien (2013) | Community clinics | Social workers | Immigrant Mexican survivors | General IPV | Qualitative, interviews | *Domestic Violence Intervention Model (DVIM)*: culturally-competent, psychoeducational, 11-week curriculum to provide IPV resources and education in a weekly group format with goals of increasing awareness, increasing self-esteem, empowering women to seek help, and increasing decision-making ability in relationships. Topics include sense of self, characteristics of healthy relationships, dynamics of IPV, and accessing resources | No | Women found the model helpful, facilitating examination of current relationships, dating, and IPV dynamics and reporting changes in self-esteem. Use of varied instructional methods (e.g., drawing, self-reflection, group discussion) was appreciated |
| McFarlane et al. (2006) | Community clinics | Nurses | IPV survivors | Physical, sexual | Quantitative, RCT | Nurse case management includes reviewing a safety planning brochure, supportive/empathic listening, anticipatory guidance (e.g., what to expect if accessing services, potential risks), and guided referrals tailored to needs | Yes | No significant differences in  reduction in violence, adoption of safety behaviors, and use of community resources between women who received a referral card vs. nurse case management protocol |
| McNamara et al. (2008) | Social service | Social workers | IPV survivors | General IPV | Quantitative, pre-post | Short-term counseling (including case management) using a feminist orientation with elements of cognitive-behavioral, existential, solution-focused, and family or systems concepts tailored to survivors’ needs | No | Women who received services significantly improved on clinical measures of life functioning, coping ability, and feeling helped and satisfied with these services |
| Parker et al. (1999) | Community clinics | Nurses | Pregnant IPV survivors | Physical, sexual, emotional | Quantitative, cohort | Empowerment protocol involving development of a safety plan, provision of information on cycle of violence, and community resources. Women were also provided a brochure with this information and were invited to attend three additional counseling sessions at a local shelter | Yes | Women who received the intervention reported significantly less violence after delivery |
| Rodgers et al. (2017) | Community clinics | Community health workers, IPV organization staff (advocates) | IPV survivors | Physical, emotional | Quantitative, RCT, qualitative, interviews | Clinic-based family health advocate who provides brief motivational intervention plus follow-up by CHW, who provides counseling on safety planning, facilitated access to IPV services and social/economic resources, and provided intensive weekly emotional and social support | No | CHWs successfully enrolled and engaged IPV survivors, who reported satisfaction and increased safety with program participation despites complex psychosocial barriers that prevented fully achieving safety and security |

Notes: RCT = randomized controlled trial; significance was reported at α = .05 level
